# Conventional swallowing training associated with electric stimulation for dysphagia following organ preservation treatment for head and neck cancer: Case report

**DOI:** 10.1016/j.bjorl.2024.101474

**Published:** 2024-07-22

**Authors:** Rafaela Negretti de Lima, Gisele Lourenço, Gustavo Jacob Lourenço, Lucia Figueiredo Mourão, Carmen Silvia Passos Lima

**Affiliations:** a Universidade Estadual de Campinas (UNICAMP), Faculdade de Ciências Médicas (FCM), Departamento de Desenvolvimento Humano e Reabilitação, Campinas, SP, Brazil; b Universidade Estadual de Campinas (UNICAMP), Faculdade de Ciências Médicas (FCM), Serviço de Oncologia Clínica, Departamento de Anestesiologia, Oncologia e Radiologia, Campinas, SP, Brazil; c Universidade Estadual de Campinas (UNICAMP), Faculdade de Ciências Médicas (FCM), Laboratório de Genética do Câncer, Campinas, SP, Brazil

## Introduction

Head and Neck Squamous Cell Carcinoma (HNSCC) is a highly incident and prevalent tumor worldwide. The tumor was the sixth most common tumor in Brazil, with 23,000 new cases each year and 11,000 annual deaths during the period of 2023‒2025. The patient’s overall survival was around 40%–50% in 5 years. The treatment of HNSCC patients with Chemoradiation (CRT) can offer a survival rate like surgical tumor resection, with the advantage of preserving organ anatomy, but dysphagia related to muscle fibrosis may arise as a deleterious effect.^1^

The effectiveness of Neuromuscular Electric Stimulation (NMES) in dysphagia of HNSCC patients treated with organ preservation regimens is uncertain. Bhatt et al. (2015)^2^ indicated NMES and Hamamoto et al. (2023)^3^ indicated Transcutaneous Electric Sensory Stimulation (TESS), which is very similar to NMES, as a supplement to reduce dysphagia in patients with HNSCC, but Langmore et al. (2016)^4^ and Van Daele et al. (2019)^5^ did not observe benefits from combining NMES with Conventional Swallowing Training (CST) in HNSCC patients undergoing CRT. We present herein an Oral Squamous Cell Carcinoma (OSCC) patient treated with CRT, who obtained improvement of dysphagia with NMES and CST.

## Case report

V. E., a 56-year-old smoker and drinker male, was diagnosed with OSCC in January 2016. The tumor was staged as T3N2M0 (AJCC, 2010). Concurrent cervicofacial fractionated radiation (2 Gy/day) for 35 days and intravenous CDDP (100 mg/m^2^ on days 1, 22, and 43) were administered to the patient resulting in complete remission of the tumor. In February 2018, the patient underwent a speech therapist evaluation due to persistent dysphagia. Computed Tomography (CT) of the head and neck and Positron Emission tomography with 2-deoxy-2-[fluorine-18]fluoro-D-glucose integrated with CT (^18^F-FDG PET/CT) were performed to confirm tumor complete remission at this time.

The patient underwent anamnesis, swallowing evaluation, subjective evaluation, Fiberoptic Endoscopic Evaluation of Swallowing **(**FEES) pre and post treatment. The Functional Oral Intake Scale (FOIS), Penetration-Aspiration Scale (PAS), Dysphagia Outcome and Severity Scale (DOSS) and Dynamic Imaging Grade of Swallowing Toxicity for Flexible Endoscopic Evaluation of Swallowing (DIGEST-FEES) were classified in both moments.

Anamnesis was conducted pre- and post-treatment, including questions about dysphagia, feeding conditions (consistency, posture, utensils, and incidents), and a history of recurrent pneumonia. General status (motor, cognitive, and communication), breathing (type and mode), orofacial organs (tone, posture, and mobility), oral reflexes, vocal quality, and saliva swallowing were considered. For the swallowing evaluation, to achieve thickened liquid and pureed consistencies, a food thickener based on xanthan gum was used. It was added to room temperature filtered water, following the consistency standardization of the product Resource ThickenUp Clear (Nestlé): thickened liquid: one measured spoon of thickener for 100 mL of water; pureed: three measured spoons of thickener for 100 mL of water. The solid consistency was achieved by offering “saltine” crackers.

The patient’s dysphagia was classified using the criteria established by the FOIS. Fiberoptic Endoscopic Evaluation of Swallowing **(**FEES), performed pre- and post-treatment using the PENTAX FNL-10RAP nasofiberscope, allowed functional observation of the pharyngeal phase of swallowing and visualization of silent aspiration. FEES was performed by an otolaryngologist offering foods colored with blue food dye. Food boluses were offered in the following sequence and in 10 mL each: 1) Slightly thickened liquid, 2) Moderately thickened liquid, 3) Solid (1/4 water biscuit), and 4) Thin liquid. The results were scored by the Penetration Aspiration Scale proposed by Rosenbek, where a higher score indicates more severe dysphagia, and the DOSS. The swallowing safety was graduated based on the DIGEST-FEES.^6^

The subjective evaluation of swallowing pre- and post-treatment used the M. D. Anderson Dysphagia Questionnaire (MDADI) and assessed the impact of dysphagia on the patient’s quality of life.^7^ According to the questionnaire’s validation protocol, the lower the quality of life-related to swallowing, the lower the scores found in each of the questionnaire’s domains: emotional (six questions), functional (five questions), and physical (eight questions). The scoring ranged from 0 to 20 indicating profound limitation, 21–40 indicating severe limitation, 41–60 indicating moderate limitation, 61–80 indicating mild limitation, and 81–100 indicating minimal limitation. The CST exercises were based on muscle strengthening for the swallowing process through direct approaches, with instructions or isotonic and sensitivity exercises, or through an indirect approach with adaptations to swallowing, following the literature.^1^, ^8^, ^9^ The selected exercises, with their respective objectives, were: 1) Tongue hold: increasing movement of the posterior pharyngeal wall during swallowing; 2) /k/ phoneme emission: increasing base of tongue contact with the posterior pharyngeal wall, favoring oral ejection; 3) Shaker maneuver: improving elevation, anteriorization, and stabilization of the hyoid larynx complex, aiming to optimize the closure of the supraglottic region, increasing the efficiency of airway protection mechanisms; and 4) Effortful swallowing: increasing the muscular strength of involved structures, optimizing bolus transport through the oropharynx.

For NMES, the Neurodyn III equipment (IBRAMED, São Paulo, Brazil) was used. Electrodes were positioned over the hyoid bone and thyroid cartilage, inferiorly and slightly medial to the posterior horn of the hyoid bone (region of the thyrohyoid muscle). Electrical stimulation was performed on dysphagic patients according to the protocol developed by Guimarães & Guimarães,^10^ named “Specific Program with Electrical Stimulation for Oropharyngeal Dysphagia”. The program consists of five stages covering muscle warm-up, enhancement of type I muscle fibers combined with resistance exercises, enhancement of type II muscle fibers combined with strength and speed exercises, stimulation of muscle tone of all muscle fibers (types I and II) through resistance, strength, and speed exercises, and finally, the cool-down phase. The parameters used in the study are described in Table 1. The treatment was administered weekly due to the patient’s mobility difficulties for ten weeks.

**Table 1:** Intervention and parameters of neuromuscular electrical stimulation associated with traditional swallowing training in patients with head and neck squamous cell carcinoma. Campinas, São Paulo, 2018.

Parameters	Exercises	Execution mode
TENS at 10 Hz, pulse width 200 µs for 3 min in warm-up; FES at 19 Hz, pulse width 250 µs for 10 min in sensorimotor mobilization concurrently with the exercise	Tongue-hold	15 repetitions
FES at 30 Hz, pulse width 300 µs with 15 contractions in the activation of type I fibers concurrently with the exercise	‒	‒
FES at 80 Hz, pulse width 300 µs with 15 contractions in the activation of type II fibers concurrently with the exercise	Production of the /k/ phoneme	15 repetitions
FES at 30 Hz, pulse width 300 µs with 15 contractions in the activation of type I fibers concurrently with the exercise.	‒	‒
FES at 80 Hz, pulse width 300 µs with 15 contractions in the activation of type II fibers concurrently with the exercise	Production of the /k/ phoneme	15 repetitions
FES at 50 Hz, pulse width 300 µs with 15 contractions in muscle toning concurrently with the exercise	Swallowing of saliva with effort	20 repetitions
TENS at 20 Hz for 5-minutes for muscle cooldown.	‒	‒
After the completion of the neuromuscular electrical stimulation	Shaker maneuver	Positioned on a stretcher, with 3 sets of 1-minute each

TENS, Transcutaneous Electrical Nerve Stimulation; FES, Functional Electrical Stimulation.

NMS with CST began about a year and a half after the end of CTR. The patient undergoing TST combined with NMES scored level 6 on the FOIS scale before and after treatment, indicating the maintenance of exclusive oral feeding with some modifications in the consistency of the ingested food. Regarding the degrees of penetration and aspiration of food into the airways, there was an improvement, particularly in swallowing more solid foods, leading to a change in the dysphagia classification from moderate to mild (Table 2). Swallowing safety significantly improves after traditional swallowing training combined with NMES (Table 3).

**Table 2:** Degrees of penetration and aspiration on the International Dysphagia Diet Standardization Initiative Inc consistencies and intensity of dysphasia pre- and post-treatment. Campinas, São Paulo, 2018.

Variable	Degrees of penetration and aspiration				Dysphagia
	Liquid	STL	MTL	Solid	
Pre-treatment	0	4	5	5	Moderate
Post-treatment	0	1	2	2	Mild

STL, Slightly Thickened Liquid; MTL, Moderately Thickened Liquid. The number “0” indicates no penetration/aspiration.

**Table 3:** Description of Digest safety degree on the International Dysphagia Diet Standardization Initiative Inc consistencies pre- and post-treatment. Campinas, São Paulo, 2018.

Variable	Dynamic Imaging Grade of Swallowing			
	Liquid	STL	MTL	Solid
Pre-treatment	0	1	2	2
Post-treatment	0	0	0	0

STL, Slightly Thickened Liquid; MTL, Moderately Thickened Liquid.

When subjective evaluation was considered, the patient showed improvement in the scores across different domains of the MDADI, and the most significant change was observed in the physical domain. In the overall calculation for the proposed classification of the questionnaire, the patient transitioned from a moderate to a minimal limitation, indicating an enhancement in the quality of life with the treatment (Table 4).

**Table 4:** The description of MD Anderson dysphagia questionnaire pre- and post-treatment. Campinas, São Paulo, 2018.

Variable	MD Anderson Dysphagia Questionnaire				
	Global	Functional	Physical	Emotion	Overall
Pre-Treatment	80	80	60	73.3	Lmed
Post-Treatment	100	88	85	93.3	Lmin

The numbers represent the scores in each domain. “Lmed” and “Lmin” stand for moderate and minimal limitation, respectively.

In the last outpatient medical consultation (June 2021), the patient did not present evidence of OSCC, or symptoms related to dysphagia.

## Discussion

Regarding the instrumental assessment of swallowing, analyzed through the DIGEST-FEES scale, signs that interfere with swallowing safety were found to be minimized when the proposed therapy was performed, reducing risks of aspiration and penetration. Improvement in airway protection was observed, resulting in reduced severity of dysphagia and increased domains of quality of life in the patient with advanced OSCC undergoing organ preservation treatment with NMES and CST. Our findings give support to results found by Blat et al. and Hamamoto et al., but oppose the results found by Langmore et al. and Van Daele et al. It is possible that these differences were determined by different protocols used in distinct studies.

## Conclusion

Our data suggest that NMES combined with CST as conducted in this study can be beneficial in managing dysphagia in patients with advanced OSCC treated with organ preservation therapy. We are aware that randomized studies with a larger number of patients treated with NMES associated with CST and with CST alone are necessary to obtain consistent conclusion about the benefit of MMS in CRT-induced dysphagia.

## Funding

None to declare.

## Conflicts of interest

The authors declare no conflicts of interest.
